# Microconfined High-Pressure Transcritical Channel Flow Database: Laminar, Transitional & Turbulent Regimes

**DOI:** 10.1038/s41597-024-03757-4

**Published:** 2024-08-23

**Authors:** Ahmed Abdellatif, Carlos Monteiro, Marc Bernades, Lluís Jofre

**Affiliations:** Department of Fluid Mechanics, Universitat Politècnica de Catalunya · BarcelonaTech (UPC), Barcelona, 08019 Spain

**Keywords:** Mechanical engineering, Energy modelling

## Abstract

The potential of comprehending and managing microscale flows to enhance energy processes, especially in heat transfer and propulsion applications, remains largely untapped particularly for supercritical fluids, which have gained increased interest over the past years due to the higher power and thermodynamic efficiencies they provide. This work, therefore, presents the first comprehensive, open-source dataset carefully curated and structured for studying microconfined high-pressure transcritical fluid channel flows under various regimes. Particularly, the dataset contains 18 direct numerical simulations of carbon dioxide at different bulk pressures and velocities confined between differentially-heated walls. For all cases, the thermodynamic conditions selected impose the fluid to undergo a transcritical trajectory across the pseudo-boiling region. The data collection comprises an array of physical quantities that enable comprehensive parametric analyses spanning laminar, transitional, and turbulent flow regimes. This data repository is poised to provide access to the detailed study and modeling of the complex flow physics observed in high-pressure transcritical fluids, especially those closely linked to improving microfluidics performance.

## Background & Summary

Over the past decades, the field of microfluidics has experienced rapid expansion driven by its extraordinary high surface-to-volume ratios, precise flow control, and well-matched length scales for interacting with microscopic elements^1^. However, typical microfluidic systems face limitations in terms of flow mixing and heat transfer due to their inherent operation under laminar flow regimes as a result of their characteristic small dimensions (*H* ∼1−1000 *μ*m) and relatively low bulk velocities (${U}_{b}\mathop{ < }\limits_{ \tilde {}}1$ m/s). Specifically, at atmospheric pressure conditions, the bulk Reynolds numbers $R{e}_{b}={U}_{b}H/{\nu }_{b}$ encountered in microfluidics fall within the approximate range 0.1–100, where the bulk kinematic viscosity ${\nu }_{b}$ typically ranges from 10^−6^ to 10^−4^ m^2^/s. In this regard, mixing within microdevices stands as one of the pivotal technologies for enabling miniaturized analysis and mass & energy transfer/conversion solutions. For instance, selected microfluidic applications in which their performance depends upon how rapidly homogeneous mixing is generated include high-throughput chemical synthesis, preparation of emulsions, protein folding, flash chemistry, and high-pressure chromatography. In this context, a crucial field of study in microfluidics revolves around enhancing molecular diffusion within laminar flows through various strategies, like the fabrication of microchannels with serpentine patterns^2^. Nonetheless, this technique frequently introduces significant complexities in manufacturing, without delivering remarkable increases in mixing and transfer rates. Alternative strategies that have been extensively investigated, for example in the works of Wang *et al*.^3^ & Nan *et al*.^4^, attempt to enhance chaotic mixing through electrokinetic forcing. This type of approaches, however, requires the utilization of fluids with significantly different electric properties. A different (brute-force) strategy is to extraordinarily increase the volumetric flow rate (large sizes/velocities) in microdevices to reach incipient turbulent flow conditions^5–7^. Although interesting for some applications, for instance high throughput liquid-liquid extraction^8^, this approach presents two main drawbacks: (i) there is no intrinsic mechanism to trigger flow destabilization, and (ii) the volumetric flow rates required are notably high in contradiction to the small rates typically sought in microfluidic applications. Finally, an interesting approach recently proposed is the utilization of cross-flow micro-jets^9^ to enhance the generation of large-scale (laminar) vortices in microfluidic systems, which aids in increasing mixing and heat transfer.

Nonetheless, a different promising strategy, which differs from conventional microfluidic practices, has been recently proposed^10,11^ based on inducing microconfined turbulence by operating under high-pressure transcritical conditions to harness the unique thermophysical properties of supercritical fluids. Supercritical fluids, which exist at pressures above the critical pressure ${P}_{c}$, undergo a complex second-order phase transition characterized by substantial property changes across the pseudo-boiling region, i.e., transcritical trajectory^10,12^. This transition, which occurs at the pseudo-boiling temperature ${T}_{pb}$ and is responsible for flow destabilization through baroclinic torque effects^13^, divides the fluid into two distinct regions marked by non-ideal behavior. Specifically, this behavior is characterized by significant deviations from the ideal-gas law, where the compressibility factor *Z* deviates from unity, caused by the modification of the molecular structure of the fluid resulting from the phase change. In detail, at temperatures below the pseudo-boiling temperature ($T < {T}_{pb}$), the fluid behaves as a supercritical liquid-like state characterized by: (i) high density *ρ*; (ii) low isothermal compressibility ${\beta }_{T}\,\mathrm{=}\,(\mathrm{1/}\rho )(\partial \rho /\partial P{)}_{T}$; and (iii) decreasing dynamic viscosity *μ* and thermal conductivity *κ* with temperature. Conversely, when the temperature exceeds the pseudo-boiling point ($T > {T}_{pb}$), the fluid is at supercritical gas-like state and exhibits opposite characteristics/behaviors. Furthermore, as discussed in previous studies^12,14^, it is worth noting that the utilization of high-pressure conditions is not limited to microfluidic research but also extends to a broad spectrum of engineering applications, such as supercritical water-cooled reactors and liquid rocket engines. This underscores the broader significance of the unconventional method discussed earlier and its potential impact on diverse technological solutions. In this regard, studying supercritical fluids experimentally poses considerable challenges, primarily because of the elevated pressures typically required, which complicate optical and sensing access. The thermophysical complexity of such systems is further heightened by the interest to operate under microfluidic conditions. Hence, the accurate characterization and prediction of high-pressure transcritical turbulent flows typically necessitate the use of computational scale-resolving approaches, such as direct numerical simulation (DNS) and large-eddy simulation (LES). Several notable examples of scale-resolving studies in the field include DNS investigations of supercritical heat transfer in pipe flows conducted by Bae *et al*.^15^, studies of transcritical flows near the critical point carried out by Sengupta *et al*.^16^, and research by Kawai^17^ involving DNS of transcritical turbulent boundary layers.

In the context of microconfined supercritical fluids, Bernades *et al*.^13^ conducted a DNS of channel flow using Nitrogen as the working fluid. Differently to the thermodynamic regimes considered in this work, the study focused only on 2 specific differentially-heated scenarios at atmospheric (ideal gas) and high-pressure (real gas) conditions. The simulations aimed to achieve a friction Reynolds number of $R{e}_{\tau }\mathrm{=100}$ at the cold/bottom wall to explore the possibility of inducing turbulence under these conditions. In this regard, motivated by the lack of sufficient research data and the escalating scientific interest on high-pressure transcritical flows at microfluidic conditions, the present work aims to carefully compute a set of DNSs to generate a curated database of 18 microconfined high-pressure transcritical channel cases encompassing laminar, transitional, and turbulent flow regimes. To that end, the numerical methods utilized for the simulations are presented, along with a comprehensive description of the framework employed for the modeling of supercritical fluids. In doing so, the first curated and logically structured computational microconfined high-pressure transcritical channel flow dataset is presented for immediate use, enabling researchers to analyze and explore the intricacies of such complex flows, along with the availability of data for the replication of computational experiments.

## Methods

The mathematical modeling utilized for studying microconfined supercritical fluids in terms of (i) equations of fluid motion, (ii) real-gas thermodynamics, (iii) high-pressure transport coefficients, and (iv) numerical methods is described below. This framework has been previously utilized to investigate the physics of high-pressure transcritical flows^11,13,18,19^, develop advanced numerical schemes^20,21^, and perform data-driven analysis^22,23^. The section culminates with an in-depth exposition of the specific computational experiments featured in the dataset.

### Equations of fluid motion

The motion of high-pressure transcritical fluids is described by the conservation of mass, momentum, and total energy, which in dimensionless form are written as

1 $$\frac{\partial {\rho }^{\star }}{\partial {t}^{\star }}+{\nabla }^{\star }\cdot ({\rho }^{\star }{{\bf{u}}}^{\star })=0,$$

2 $$\frac{\partial ({\rho }^{\star }{{\bf{u}}}^{\star })}{\partial {t}^{\star }}+{\nabla }^{\star }\cdot ({\rho }^{\star }{{\bf{u}}}^{\star }{{\bf{u}}}^{\star })=-{\nabla }^{\star }{P}^{\star }+\frac{{\nabla }^{\star }\cdot {\tau }^{\star }}{R{e}_{b}},$$

3 $$\frac{\partial ({\rho }^{\star }{E}^{\star })}{\partial {t}^{\star }}+{\nabla }^{\star }\cdot ({\rho }^{\star }{{\bf{u}}}^{\star }{E}^{\star })=\frac{{\nabla }^{\star }\cdot ({\kappa }^{\star }{\nabla }^{\star }{T}^{\star })}{R{e}_{b}P{r}_{b}E{c}_{b}}-{\nabla }^{\star }\cdot ({P}^{\star }{{\bf{u}}}^{\star })+\frac{{\nabla }^{\star }\cdot ({\tau }^{\star }\cdot {{\bf{u}}}^{\star })}{R{e}_{b}},$$

where superscript $\star$ denotes normalized quantities, *t* is the time, **u** is the velocity vector, *ρ* is the density, *P* is the pressure, $\tau =\mu (\nabla {\bf{u}}+{\nabla }^{\star }{\bf{u}})-\mathrm{(2}\mu \mathrm{/3)(}\nabla \cdot {\bf{u}}){\bf{I}}$ is the viscous stress tensor with *μ* the dynamic viscosity and **I** the identity matrix, $E=e+|{\bf{u}}{|}^{2}\mathrm{/2}$ and *e* are the total and internal energy, respectively, *T* is the temperature, and *κ* is the thermal conductivity. The obtention of these dimensionless equations is fundamented on the following set of inertial-based scalings^23,24^

4 $${{\bf{x}}}^{\star }=\frac{{\bf{x}}}{H},\,{{\bf{u}}}^{\star }=\frac{{\bf{u}}}{{U}_{b}},\,{\rho }^{\star }=\frac{\rho }{{\rho }_{b}},\,{T}^{\star }=\frac{T}{{T}_{b}},\,{P}^{\star }=\frac{P}{{\rho }_{b}{U}_{b}^{2}},\,{E}^{\star }=\frac{E}{{U}_{b}^{2}},\,{\mu }^{\star }=\frac{\mu }{{\mu }_{b}},\,{\kappa }^{\star }=\frac{\kappa }{{\kappa }_{b}},$$

with subscript *b* indicating bulk quantities, **x** the position vector, *H* the total channel height, and *U* the time-averaged streamwise velocity. The resulting set of scaled equations includes three dimensionless numbers: (i) bulk Reynolds number $R{e}_{b}={\rho }_{b}{U}_{b}H/{\mu }_{b}$ characterizing the ratio between inertial and viscous forces; (ii) bulk Prandtl number $P{r}_{b}={\mu }_{b}{c}_{Pb}/{\kappa }_{b}$, where *c*_*P*_ is the isobaric heat capacity, quantifying the ratio between momentum and thermal diffusivity; and (iii) bulk Eckert number $E{c}_{b}={U}_{b}^{2}/({c}_{Pb}{T}_{b})$ accounting for the ratio between advective mass transfer and heat dissipation potential.

### Real-gas thermodynamics

The thermodynamic space of solutions for the state variables pressure *P*, temperature *T*, and density *ρ* of a monocomponent fluid is described by an equation of state. In this work, therefore, the Peng-Robinson equation of state^25^, which is suitable for high-pressure thermodynamic conditions as provided in the work by Jofre & Urzay^12^, is utilized to close the equations of fluid motion above. In general form, it can be expressed in terms of the compressibility factor $Z=P/(\rho {R}^{{\rm{{\prime} }}}T)$ with ${R}^{{\rm{{\prime} }}}=188.9\,{\rm{J}}/({\rm{k}}{\rm{g}}\cdot {\rm{K}})$ the specific gas constant of CO_2_, which in dimensionless form is written as

5 $${P}^{\star }=\frac{Z{\rho }^{\star }{T}^{\star }}{{\hat{\gamma }}_{b}M{a}_{b}^{2}},$$

where $\hat{\gamma }\approx Z({c}_{P}/{c}_{V})[(Z+T{(\partial Z/\partial T)}_{\rho })/(Z+T{(\partial Z/\partial T)}_{P})]$ is an approximated real-gas heat capacity ratio^26^ with *c*_*V*_ the isochoric heat capacity. As it can be noted, the dimensionless bulk Mach number $M{a}_{b}={U}_{b}/{c}_{b}$ appears, where *c*_*b*_ is the bulk speed of sound, which represents the ratio of flow velocity to the local speed of sound. In addition, real-gas equations of state need to be supplemented with the corresponding high-pressure thermodynamic variables (e.g., internal energy, heat capacities) based on departure functions^27^ calculated as a difference between two states. In particular, their usefulness is to transform thermodynamic variables from ideal-gas conditions (low pressure - only temperature dependant) to supercritical conditions (high pressure). The ideal-gas parts are calculated by means of the NASA 7-coefficient polynomial^28^, while the analytical departure expressions to high pressures are derived from the Peng-Robinson equation of state^12^.

### High-pressure transport coefficients

The high pressures involved in the analyses conducted in this work prevent the use of simple relations for the calculation of dynamic viscosity *μ* and thermal conductivity *κ*. In this regard, standard methods for computing these coefficients for Newtonian fluids are based on the correlation expressions proposed by Chung *et al*.^29,30^. These correlation expressions are mainly function of critical temperature *T*_*c*_ and density *ρ*_*c*_, molecular weight *W*, acentric factor *ω*, association factor *κ*_*a*_ and dipole moment ${\mathscr{M}}$, and the NASA 7-coefficient polynomial^28^; further details can be found in dedicated works, like for example Poling *et al*.^31^ and Jofre and Urzay^12^.

### Numerical method

The equations of fluid motion are computationally solved by means of the in-house flow solver RHEA^32^. A standard semi-discretization procedure is adopted in which they are firstly discretized in space and then integrated in time. In particular, spatial operators are treated using second-order central-differencing schemes, and time-advancement is explicitly performed by means of a third-order strong-stability preserving (SSP) Runge-Kutta approach^33^ coupled with an artificial compressibility method (ACM)^34^. The convective terms are expanded according to the Kennedy-Gruber-Pirozzoli (KGP) splitting^35,36^, which has been recently extended to high-pressure transcritical fluids turbulence in previous works^20,37,38^. The method preserves kinetic energy by convection, and is locally conservative for mass, momentum, and total energy. This numerical framework provides stable computations without the need of any form of artificial dissipation or stabilization procedures.

### Computational experiments

**Fig. 1:**
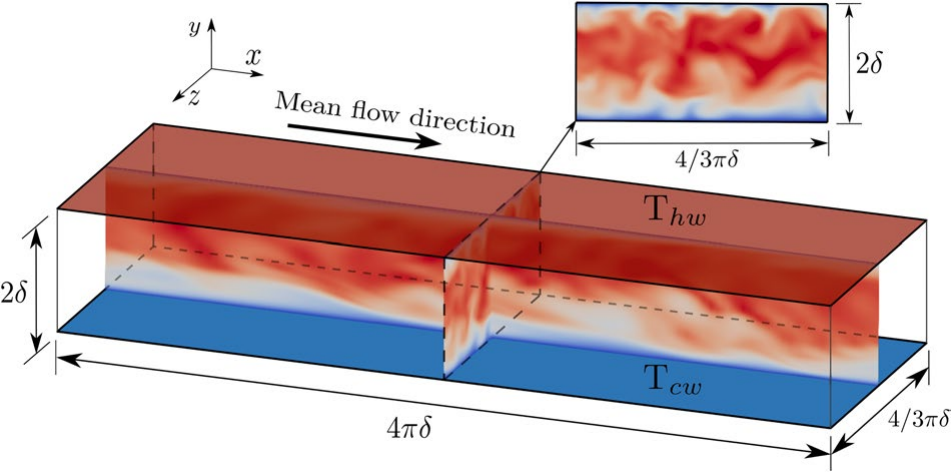
Schematic of a microconfined channel flow domain with cold (*cw*) and hot (*hw*) isothermal walls. The illustration also shows the domain dimensions and the mean flow direction.

**Fig. 2:**
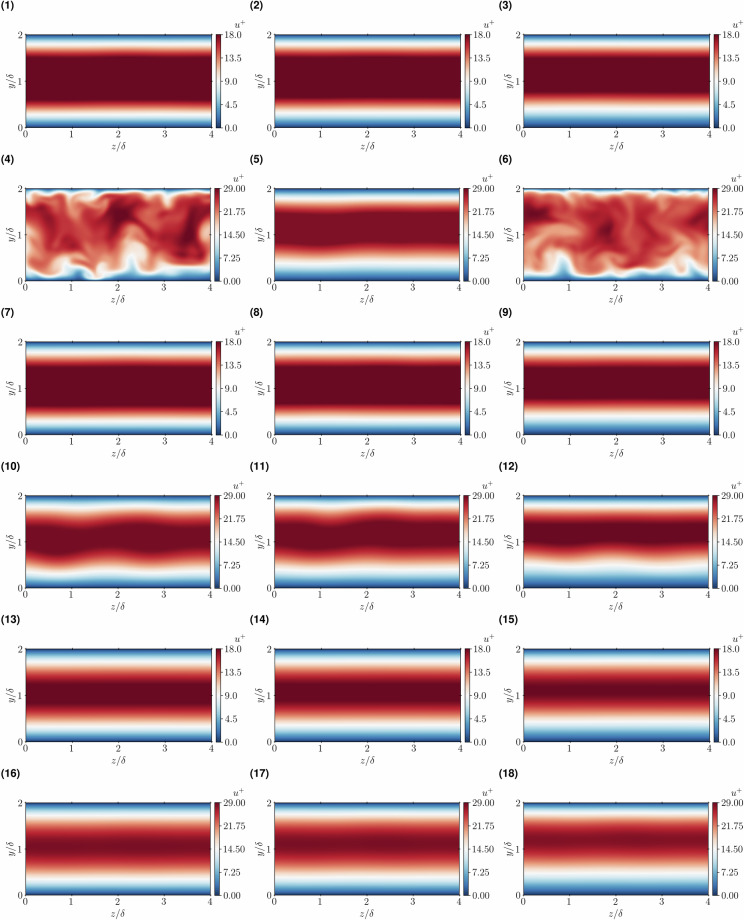
Snapshots of instantaneous streamwise velocity in wall units *μ*^+^ on a $z/\delta$-$y/\delta$ slice for the 18 cases.

As portrayed in Fig. 1, a high-pressure transcritical microfluidic channel flow at low-Mach-number conditions is chosen for the dataset generation. The selected fluid is CO_2_ with its critical thermophysical conditions and properties outlined in Table 1. The microchannel is operated at three distinctive supercritical bulk pressures ${P}_{b}/{P}_{c}=1.5$, 2 and 5, with the fluid enclosed between two parallel isothermal walls at cold (*cw*) and hot (*hw*) temperatures expressed as *T*_*cw*_/*T*_*c*_ and *T*_*hw*_/*T*_*c*_, respectively. These temperature values lie within the specified intervals Δ*T*/*T*_*c*_ = (*T*_*hw*_ − *T*_*cw*_)/*T*_*c*_ = 0.8–1.4, 0.9–1.2 and 0.95–1.1. The separation between walls is equal to *H* = 2*δ*, with *δ* = 100 *μm* representing half of the channel height. The fluid flows from left to right in the streamwise direction with bulk velocities *U*_*b*_ = 0.5 and 1 *m*/*s*, which is imposed through a body force controlled by a proportional feedback loop with gain *k*_*p*_ = 0.1 aimed at reducing the difference between the desired and measured (numerical) values; the relative errors achieved are below 0.5% for all cases. This range of parameters compels the fluid to undergo varying flow conditions as it traverses the pseudo-boiling line through a transcritical thermodynamic trajectory^13,22^. In particular, as detailed in Table 2 and qualitatively represented in Fig. 2, the resulting dataset comprises a total of 18 simulations encompassing laminar (non-symmetric), transitional and turbulent flow regimes^39^. It is important to highlight that, although it is difficult to observe from the streamwise velocity colormaps of Fig. 2, none of the cases presented are trivially symmetric as they present spanwise oscillations/fluctuations of flow quantities and fluid properties. In detail, the baroclinic-type instabilities generated at the pseudo-boiling region modulate the flow in the three directions, and consequently no complete symmetry is observed for any case.

**Table 1:** Critical pressure, temperature and density, molecular weight and acentric factor for CO_2_ from NIST data^44^.

Fluid	*P*_*c*_[MPa]	*T*_*c*_[K]	*ρ*_*c*_[kg/m^3^]	*W*[kg/mol]	*ω*
CO_2_	7.38	304.13	467.60	0.04401	0.0372

**Table 2:** Input parameters and dimensionless numbers of the database cases.

No.	*P*_*b*_/*P*_*c*_	*T*_*cw*_/*T*_*c*_	*T*_*hw*_/*T*_*c*_	*U*_*b*_ [m/s]	*Re*_*b*_	*Pr*_*b*_	*Ec*_*b*_	*Ma*_*b*_
1	1.5	0.95	1.1	0.50	1168	2.76	2.08E-07	0.015
2	1.5	0.90	1.2	0.50	1059	2.28	2.56E-07	0.014
3	1.5	0.80	1.4	0.50	843	1.85	3.27E-07	0.013
4	1.5	0.95	1.1	1.00	2385	2.83	8.16E-07	0.029
5	1.5	0.90	1.2	1.00	2108	2.27	1.04E-06	0.029
6	1.5	0.80	1.4	1.00	2030	2.34	1.06E-06	0.024
7	2.0	0.95	1.1	0.50	1108	2.06	2.77E-07	0.012
8	2.0	0.90	1.2	0.50	1047	2.00	2.87E-07	0.013
9	2.0	0.80	1.4	0.50	857	1.77	3.35E-07	0.012
10	2.0	0.95	1.1	1.00	2221	2.05	1.13E-06	0.025
11	2.0	0.90	1.2	1.00	2084	2.01	1.17E-06	0.025
12	2.0	0.80	1.4	1.00	1693	1.77	1.37E-06	0.024
13	5.0	0.95	1.1	0.50	832	1.41	4.25E-07	0.008
14	5.0	0.90	1.2	0.50	835	1.41	4.16E-07	0.009
15	5.0	0.80	1.4	0.50	775	1.45	4.12E-07	0.009
16	5.0	0.95	1.1	1.00	1666	1.41	1.71E-06	0.017
17	5.0	0.90	1.2	1.00	1676	1.41	1.70E-06	0.017
18	5.0	0.80	1.4	1.00	1531	1.45	1.67E-06	0.017

The computational domain is $4\pi \delta \times 2\delta \times \mathrm{(4/3)}\pi \delta$ in the streamwise (*x*), wall-normal (*y*), and spanwise (*z*) directions, respectively, which is large enough to represent the largest flow scales of the problem^40^. The streamwise and spanwise boundaries are set periodic, and no-slip conditions are imposed on the horizontal boundaries (*x*-*z* planes). The resulting DNS grid contains 96 × 96 × 96 points uniformly distributed in the streamwise and spanwise directions. In particular, the mesh resolutions in *cw*-units fall within the ranges $2.70\mathop{ < }\limits_{ \tilde {}}\Delta {x}^{+}\mathop{ < }\limits_{ \tilde {}}6.10$, $0.10\mathop{ < }\limits_{ \tilde {}}\Delta {y}^{+}\mathop{ < }\limits_{ \tilde {}}0.16$ and $0.87\mathop{ < }\limits_{ \tilde {}}\Delta {z}^{+}\mathop{ < }\limits_{ \tilde {}}2.10$ (using *hw*-units the resolutions correspond to $8.3\mathop{ < }\limits_{ \tilde {}}\Delta {x}^{+}\mathop{ < }\limits_{ \tilde {}}10.1$, $0.31\mathop{ < }\limits_{ \tilde {}}\Delta {y}^{+}\mathop{ < }\limits_{ \tilde {}}0.40$ and $4.17\mathop{ < }\limits_{ \tilde {}}\Delta {z}^{+}\mathop{ < }\limits_{ \tilde {}}5.10$), while the mesh is stretched towards the walls in the wall-normal direction such that the first grid point is at ${y}_{cw}^{+}=y{u}_{\tau ,cw}/{\nu }_{cw}\mathop{ < }\limits_{ \tilde {}}0.05$ from the cold wall and ${y}_{hw}^{+}\mathop{ < }\limits_{ \tilde {}}0.15$ from the hot wall, with the latter being the most critical one; extensive details of the resolutions in wall units can be found in the technical validation section. For the temporal time stepping, a Courant-Friedrichs-Lewy number of *CFL* = 0.1 is selected. For each case, the computational strategy starts from a linear velocity profile with random fluctuations^41^, which is advanced in time for approximately 6 and 11 flow-through-time (FTT) units for the *U*_*b*_ = 0.5 m/s and *U*_*b*_ = 1 m/s cases, respectively, after achieving statistically steady-state flow conditions (approximately 10 FTTs); based on the bulk velocity *U*_*b*_ and the length of the channel ${L}_{x}=4\pi \delta$, a FTT is defined as ${t}_{FTT}={L}_{x}/{U}_{b}$.

## Data Records

The dataset is available online on Figshare^40^, a commonly used platform for sharing large datasets. The data is structured in a manner where individual cases are allocated in dedicated folders, facilitating in this way convenient access and data processing. Each case folder is structured in different FTT subfolders, which contain 10 snapshot files of the corresponding FTT uniformly spaced in time. Additionally, there is a README.txt file in each case folder that provides essential information about the individual case, such as (i) key independent parameters, (ii) dimensionless numbers, (iii) details on the calculation of the stretching factor in the y-direction, and (iv) the time advancement technique. The dataset files within the FTT subfolders are stored in the Hierarchical Data Format version 5 (h5)^42^, an open-source file format capable of handling complex and diverse data. There is also an associated xdmf file that contains data descriptions for these files, which can be visualized with, for example, the open-source software Paraview^43^. Finally, each case folder also includes a configuration file and a substances library file in YAML format. Likewise, there are C++ files in both .cpp and .hpp formats, allowing users to replicate the simulations for further investigation and/or verification.

The dataset includes an extensive array of input features and corresponding labels, as outlined in Table 2. It has been meticulously organized to facilitate the extraction of two distinct categories of data: (i) base output variables, and (ii) derived quantities. The base output variables entail critical parameters that are directly generated by the simulations, serving as essential prerequisites for the computation of other derived quantities. These base outputs primarily consist of instantaneous flow parameters. In contrast, the derived quantities comprise various statistical measures, including first- and second-order Reynolds- and Favre-averaged statistical quantities. For a given quantity, denoted as $\phi$, the Reynolds-averaged mean $\bar{\phi }$ and its fluctuation ${\phi }^{{\rm{{\prime} }}}$ are defined as $\phi =\bar{\phi }+{\phi }^{{\rm{{\prime} }}}$, with the condition that the average of $\bar{{\phi }^{{\rm{{\prime} }}}}$ equals zero. Similarly, the Favre-averaged mean $\mathop{\phi }\limits^{ \sim }$ and its fluctuation ${\phi }^{{\rm{{\prime} }}{\rm{{\prime} }}}$ are defined as $\phi =\mathop{\phi }\limits^{ \sim }+{\phi }^{{\rm{{\prime} }}{\rm{{\prime} }}}$ with $\mathop{\phi }\limits^{ \sim }=\overline{\rho \phi }/\bar{\rho }$. A summary of the primary base output fields available within the dataset is provided in Table 3, while Tables 4, 5, and 6 furnish concise overviews of the derived output fields.

**Table 3:** Base output quantities of the database cases.

Description	RHEA Quantity	Symbology	Units
Mesh coordinate in x-direction	x	*x*	m
Mesh coordinate in y-direction	y	*y*	m
Mesh coordinate in z-direction	z	*z*	m
Instantaneous streamwise velocity	u	*μ*	m/s
Instantaneous wall-normal velocity	v	*v*	m/s
Instantaneous spanwise velocity	w	*w*	m/s
Total energy	E	*E*	J/kg
Pressure	P	*P*	Pa
Temperature	T	*T*	K
Thermal conductivity	kappa	*κ*	W/(m·K)
Dynamic viscosity	mu	*μ*	Pa s
Density	rho	*ρ*	kg/m^3^
Speed of sound	sos	*c*	m/s
Specific isochoric heat capacity	c_v	*c*_*v*_	J/(kg·K)
Specific isobaric heat capacity	c_P	*c*_*P*_	J/(kg·K)

**Table 4:** Derived time-averaged quantities of the database cases.

Description	Field name	Symbology	Units	Expression
Time-averaged density	avg_rho	$\overline{\rho }$	kg/m^3^	$\overline{\rho }=\frac{1}{\varDelta t}{\int }_{0}^{\varDelta t}\rho (t)\,dt$
Time-averaged conserved streamwise momentum	avg_rhou	$\overline{\rho u}$	kg/(s·m^2^)	$\overline{\rho u}=\frac{1}{\varDelta t}{\int }_{0}^{\varDelta t}\rho u(t)\,dt$
Time-averaged conserved wall-normal momentum	avg_rhov	$\overline{\rho v}$	kg/(s·m^2^)	$\overline{\rho v}=\frac{1}{\varDelta t}{\int }_{0}^{\varDelta t}\rho v(t)\,dt$
Time-averaged conserved spanwise momentum	avg_rhow	$\overline{\rho w}$	kg/(s·m^2^)	$\overline{\rho w}=\frac{1}{\varDelta t}{\int }_{0}^{\varDelta t}\rho w(t)\,dt$
Time-averaged conserved total energy	avg_rhoE	$\overline{\rho E}$	J/m^3^	$\overline{\rho E}=\frac{1}{\varDelta t}{\int }_{0}^{\varDelta t}\rho E(t)\,dt$
Time-averaged conserved pressure	avg_rhoP	$\overline{\rho P}$	Pa·kg/m^3^	$\overline{\rho P}=\frac{1}{\varDelta t}{\int }_{0}^{\varDelta t}\rho P(t)\,dt$
Time-averaged conserved temperature	avg_rhoT	$\overline{\rho T}$	K·kg/m^3^	$\overline{\rho T}=\frac{1}{\varDelta t}{\int }_{0}^{\varDelta t}\rho T(t)\,dt$
Time-averaged streamwise velocity	avg_u	$\overline{u}$	m/s	$\overline{u}=\frac{1}{\varDelta t}{\int }_{0}^{\varDelta t}u(t)\,dt$
Time-averaged wall-normal velocity	avg_v	$\overline{v}$	m/s	$\overline{v}=\frac{1}{\varDelta t}{\int }_{0}^{\varDelta t}v(t)\,dt$
Time-averaged spanwise velocity	avg_w	$\overline{w}$	m/s	$\overline{w}=\frac{1}{\varDelta t}{\int }_{0}^{\varDelta t}w(t)\,dt$
Time-averaged total energy	avg_E	$\overline{E}$	J/m^3^	$\overline{E}=\frac{1}{\varDelta t}{\int }_{0}^{\varDelta t}E(t)\,dt$
Time-averaged pressure	avg_P	$\overline{P}$	Pa	$\overline{P}=\frac{1}{\varDelta t}{\int }_{0}^{\varDelta t}P(t)\,dt$
Time-averaged temperature	avg_T	$\overline{T}$	K	$\overline{T}=\frac{1}{\varDelta t}{\int }_{0}^{\varDelta t}T(t)\,dt$
Time-averaged speed of sound	avg_sos	$\overline{c}$	m/s	$\overline{c}=\frac{1}{\varDelta t}{\int }_{0}^{\varDelta t}c(t)\,dt$
Time-averaged dynamic viscosity	avg_mu	$\overline{\mu }$	Pa·s	$\overline{\mu }=\frac{1}{\varDelta t}{\int }_{0}^{\varDelta t}\mu (t)\,dt$
Time-averaged thermal conductivity	avg_kappa	$\overline{\kappa }$	W/(m·K)	$\overline{\kappa }=\frac{1}{\varDelta t}{\int }_{0}^{\varDelta t}\kappa (t)\,dt$
Time-averaged specific isochoric heat capacity	avg_c_v	$\overline{{c}_{v}}$	J/(kg·K)	$\overline{{c}_{v}}=\frac{1}{\varDelta t}{\int }_{0}^{\varDelta t}{c}_{v}(t)\,dt$
Time-averaged specific isobaric heat capacity	avg_c_P	$\overline{{c}_{P}}$	J/(kg·K)	$\overline{{c}_{P}}=\frac{1}{\varDelta t}{\int }_{0}^{\varDelta t}{c}_{P}(t)\,dt$

**Table 5:** Derived Reynolds-averaged root-mean-square fluctuations of the database cases.

Description	Field name	Symbology	Units	Expression
Root-mean-square density fluctuations	rmsf_rho	${\rho }_{{\rm{rms}}}$	kg/m^3^	${\rho }_{{\rm{r}}{\rm{m}}{\rm{s}}}=\sqrt{\bar{{{\rho }^{{\rm{{\prime} }}}}^{2}}}$, where $\bar{{{\rho }^{{\rm{{\prime} }}}}^{2}}=\frac{1}{\Delta t}{\int }_{0}^{\Delta t}{(\rho (t)-\bar{\rho })}^{2}\,dt$
Root-mean-square conserved streamwise momentum fluctuations	rmsf_rhou	${(\rho u)}_{{\rm{rms}}}$	kg/(s·m^2^)	${(\rho u)}_{{\rm{r}}{\rm{m}}{\rm{s}}}=\sqrt{\bar{{({\rho }^{{\rm{{\prime} }}}{u}^{{\rm{{\prime} }}})}^{2}}}$, where $\bar{{({\rho }^{{\rm{{\prime} }}}{u}^{{\rm{{\prime} }}})}^{2}}=\frac{1}{\Delta t}{\int }_{0}^{\Delta t}{(\rho u(t)-\overline{\rho u})}^{2}\,dt$
Root-mean-square conserved wall-normal momentum fluctuations	rmsf_rhov	${(\rho v)}_{{\rm{rms}}}$	kg/(s·m^2^)	${(\rho v)}_{{\rm{r}}{\rm{m}}{\rm{s}}}=\sqrt{\bar{{({\rho }^{{\rm{{\prime} }}}{v}^{{\rm{{\prime} }}})}^{2}}}$, where $\bar{{({\rho }^{{\rm{{\prime} }}}{v}^{{\rm{{\prime} }}})}^{2}}=\frac{1}{\Delta t}{\int }_{0}^{\Delta t}{(\rho v(t)-\overline{\rho v})}^{2}\,dt$
Root-mean-square conserved spanwise momentum fluctuations	rmsf_rhow	${(\rho w)}_{{\rm{rms}}}$	kg/(s·m^2^)	${(\rho w)}_{{\rm{r}}{\rm{m}}{\rm{s}}}=\sqrt{\bar{{({\rho }^{{\rm{{\prime} }}}{w}^{{\rm{{\prime} }}})}^{2}}}$, where $\bar{{({\rho }^{{\rm{{\prime} }}}{w}^{{\rm{{\prime} }}})}^{2}}=\frac{1}{\Delta t}{\int }_{0}^{\Delta t}{(\rho w(t)-\overline{\rho w})}^{2}\,dt$
Root-mean-square conserved total energy fluctuations	rmsf_rhoE	${(\rho E)}_{{\rm{rms}}}$	J/m^2^	${(\rho E)}_{{\rm{r}}{\rm{m}}{\rm{s}}}=\sqrt{\bar{{({\rho }^{{\rm{{\prime} }}}{E}^{{\rm{{\prime} }}})}^{2}}}$, where $\bar{{({\rho }^{{\rm{{\prime} }}}{E}^{{\rm{{\prime} }}})}^{2}}=\frac{1}{\Delta t}{\int }_{0}^{\Delta t}{(\rho E(t)-\overline{\rho E})}^{2}\,dt$
Root-mean-square streamwise velocity fluctuations	rmsf_u	${u}_{{\rm{rms}}}$	m/s	${u}_{{\rm{r}}{\rm{m}}{\rm{s}}}=\sqrt{\bar{{{u}^{{\rm{{\prime} }}}}^{2}}}$, where $\bar{{{u}^{{\rm{{\prime} }}}}^{2}}=\frac{1}{\Delta t}{\int }_{0}^{\Delta t}{(u(t)-\bar{u})}^{2}\,dt$
Root-mean-square wall-normal velocity fluctuations	rmsf_v	${v}_{{\rm{rms}}}$	m/s	${v}_{{\rm{r}}{\rm{m}}{\rm{s}}}=\sqrt{\bar{{{v}^{{\rm{{\prime} }}}}^{2}}}$, where $\bar{{{v}^{{\rm{{\prime} }}}}^{2}}=\frac{1}{\Delta t}{\int }_{0}^{\Delta t}{(v(t)-\bar{v})}^{2}\,dt$
Root-mean-square spanwise velocity fluctuations	rmsf_w	${w}_{{\rm{rms}}}$	m/s	${w}_{{\rm{r}}{\rm{m}}{\rm{s}}}=\sqrt{\bar{{{w}^{{\rm{{\prime} }}}}^{2}}}$, where $\bar{{{w}^{{\rm{{\prime} }}}}^{2}}=\frac{1}{\Delta t}{\int }_{0}^{\Delta t}{(w(t)-\bar{w})}^{2}\,dt$
Root-mean-square total energy fluctuations	rmsf_E	${E}_{{\rm{rms}}}$	J/kg	${E}_{{\rm{r}}{\rm{m}}{\rm{s}}}=\sqrt{\bar{{{E}^{{\rm{{\prime} }}}}^{2}}}$, where $\bar{{{E}^{{\rm{{\prime} }}}}^{2}}=\frac{1}{\Delta t}{\int }_{0}^{\Delta t}{(E(t)-\bar{E})}^{2}\,dt$
Root-mean-square pressure fluctuations	rmsf_P	${P}_{{\rm{rms}}}$	Pa	${P}_{{\rm{r}}{\rm{m}}{\rm{s}}}=\sqrt{\bar{{{P}^{{\rm{{\prime} }}}}^{2}}}$, where $\bar{{{P}^{{\rm{{\prime} }}}}^{2}}=\frac{1}{\Delta t}{\int }_{0}^{\Delta t}{(P(t)-\bar{P})}^{2}\,dt$
Root-mean-square temperature fluctuations	rmsf_T	${T}_{{\rm{rms}}}$	K	${T}_{{\rm{r}}{\rm{m}}{\rm{s}}}=\sqrt{\bar{{{T}^{{\rm{{\prime} }}}}^{2}}}$, where $\bar{{{T}^{{\rm{{\prime} }}}}^{2}}=\frac{1}{\Delta t}{\int }_{0}^{\Delta t}{(T(t)-\bar{T})}^{2}\,dt$
Root-mean-square speed of sound fluctuations	rmsf_sos	${c}_{{\rm{rms}}}$	kg/(s·m^2^)	${c}_{{\rm{r}}{\rm{m}}{\rm{s}}}=\sqrt{\bar{{{c}^{{\rm{{\prime} }}}}^{2}}}$, where $\bar{{{c}^{{\rm{{\prime} }}}}^{2}}=\frac{1}{\Delta t}{\int }_{0}^{\Delta t}{(c(t)-\bar{c})}^{2}\,dt$
Root-mean-square dynamic viscosity fluctuations	rmsf_mu	${\mu }_{{\rm{rms}}}$	Pa·s	${\mu }_{{\rm{r}}{\rm{m}}{\rm{s}}}=\sqrt{\bar{{{\mu }^{{\rm{{\prime} }}}}^{2}}}$, where $\bar{{{\mu }^{{\rm{{\prime} }}}}^{2}}=\frac{1}{\Delta t}{\int }_{0}^{\Delta t}{(\mu (t)-\bar{\mu })}^{2}\,dt$
Root-mean-square thermal conductivity fluctuations	rmsf_kappa	${\kappa }_{{\rm{rms}}}$	W/(m·K)	${\kappa }_{{\rm{r}}{\rm{m}}{\rm{s}}}=\sqrt{\bar{{{\kappa }^{{\rm{{\prime} }}}}^{2}}}$, where $\bar{{{\kappa }^{{\rm{{\prime} }}}}^{2}}=\frac{1}{\Delta t}{\int }_{0}^{\Delta t}{(\kappa (t)-\bar{\kappa })}^{2}\,dt$
Root-mean-square specific isochoric heat capacity fluctuations	rmsf_c_v	${c}_{{v}_{{\rm{rms}}}}$	J/(kg·K)	${c}_{{v}_{{\rm{r}}{\rm{m}}{\rm{s}}}}=\sqrt{\bar{{{{c}_{v}}^{{\rm{{\prime} }}}}^{2}}}$, where $\bar{{{{c}_{v}}^{{\rm{{\prime} }}}}^{2}}=\frac{1}{\Delta t}{\int }_{0}^{\Delta t}{({c}_{v}(t)-\bar{{c}_{v}})}^{2}\,dt$
Root-mean-square specific isobaric heat capacity fluctuations	rmsf_c_P	${c}_{{P}_{{\rm{rms}}}}$	J/(kg·K)	${c}_{{P}_{{\rm{r}}{\rm{m}}{\rm{s}}}}=\sqrt{\bar{{{{c}_{P}}^{{\rm{{\prime} }}}}^{2}}}$, where $\bar{{{{c}_{P}}^{{\rm{{\prime} }}}}^{2}}=\frac{1}{\Delta t}{\int }_{0}^{\Delta t}{({c}_{P}(t)-\bar{{c}_{P}})}^{2}\,dt$

**Table 6:** Derived Favre-averaged fluctuations of the database cases.

Description	Field name	Symbology	Units	Expression
Favre-averaged streamwise velocity squared fluctuations	favre_uffuff	$\mathop{{u}^{{\rm{{\prime} }}{\rm{{\prime} }}}{u}^{{\rm{{\prime} }}{\rm{{\prime} }}}}\limits^{ \sim }$	m^2^/s^2^	$\mathop{{u}^{{\rm{{\prime} }}{\rm{{\prime} }}}{u}^{{\rm{{\prime} }}{\rm{{\prime} }}}}\limits^{ \sim }=\bar{\rho {u}^{{\rm{{\prime} }}{\rm{{\prime} }}}{u}^{{\rm{{\prime} }}{\rm{{\prime} }}}}/\bar{\rho }$
Favre-averaged streamwise and wall-normal velocity fluctuations	favre_uffvff	$\mathop{{u}^{{\rm{{\prime} }}{\rm{{\prime} }}}{v}^{{\rm{{\prime} }}{\rm{{\prime} }}}}\limits^{ \sim }$	m^2^/s^2^	$\mathop{{u}^{{\rm{{\prime} }}{\rm{{\prime} }}}{v}^{{\rm{{\prime} }}{\rm{{\prime} }}}}\limits^{ \sim }=\bar{\rho {u}^{{\rm{{\prime} }}{\rm{{\prime} }}}{v}^{{\rm{{\prime} }}{\rm{{\prime} }}}}/\bar{\rho }$
Favre-averaged streamwise and spanwise velocity fluctuations	favre_uffwff	$\mathop{{u}^{{\rm{{\prime} }}{\rm{{\prime} }}}{w}^{{\rm{{\prime} }}{\rm{{\prime} }}}}\limits^{ \sim }$	m^2^/s^2^	$\mathop{{u}^{{\rm{{\prime} }}{\rm{{\prime} }}}{w}^{{\rm{{\prime} }}{\rm{{\prime} }}}}\limits^{ \sim }=\bar{\rho {u}^{{\rm{{\prime} }}{\rm{{\prime} }}}{w}^{{\rm{{\prime} }}{\rm{{\prime} }}}}/\bar{\rho }$
Favre-averaged wall-normal velocity squared fluctuations	favre_vffvff	$\mathop{{v}^{{\rm{{\prime} }}{\rm{{\prime} }}}{v}^{{\rm{{\prime} }}{\rm{{\prime} }}}}\limits^{ \sim }$	m^2^/s^2^	$\mathop{{v}^{{\rm{{\prime} }}{\rm{{\prime} }}}{v}^{{\rm{{\prime} }}{\rm{{\prime} }}}}\limits^{ \sim }=\bar{\rho {v}^{{\rm{{\prime} }}{\rm{{\prime} }}}{v}^{{\rm{{\prime} }}{\rm{{\prime} }}}}/\bar{\rho }$
Favre-averaged wall-normal and spanwise velocity fluctuations	favre_vffwff	$\mathop{{v}^{{\rm{{\prime} }}{\rm{{\prime} }}}{w}^{{\rm{{\prime} }}{\rm{{\prime} }}}}\limits^{ \sim }$	m^2^/s^2^	$\mathop{{v}^{{\rm{{\prime} }}{\rm{{\prime} }}}{w}^{{\rm{{\prime} }}{\rm{{\prime} }}}}\limits^{ \sim }=\bar{\rho {v}^{{\rm{{\prime} }}{\rm{{\prime} }}}{w}^{{\rm{{\prime} }}{\rm{{\prime} }}}}/\bar{\rho }$
Favre-averaged spanwise velocity squared fluctuations	favre_wffwff	$\mathop{{w}^{{\rm{{\prime} }}{\rm{{\prime} }}}{w}^{{\rm{{\prime} }}{\rm{{\prime} }}}}\limits^{ \sim }$	m^2^/s^2^	$\mathop{{w}^{{\rm{{\prime} }}{\rm{{\prime} }}}{w}^{{\rm{{\prime} }}{\rm{{\prime} }}}}\limits^{ \sim }=\bar{\rho {w}^{{\rm{{\prime} }}{\rm{{\prime} }}}{w}^{{\rm{{\prime} }}{\rm{{\prime} }}}}/\bar{\rho }$
Favre-averaged streamwise velocity and total energy fluctuations	favre_uffEff	$\mathop{{u}^{{\rm{{\prime} }}{\rm{{\prime} }}}{E}^{{\rm{{\prime} }}{\rm{{\prime} }}}}\limits^{ \sim }$	J·m/(kg·s)	$\mathop{{u}^{{\rm{{\prime} }}{\rm{{\prime} }}}{E}^{{\rm{{\prime} }}{\rm{{\prime} }}}}\limits^{ \sim }=\bar{\rho {u}^{{\rm{{\prime} }}{\rm{{\prime} }}}{E}^{{\rm{{\prime} }}{\rm{{\prime} }}}}/\bar{\rho }$
Favre-averaged wall-normal velocity and total energy fluctuations	favre_vffEff	$\mathop{{v}^{{\rm{{\prime} }}{\rm{{\prime} }}}{E}^{{\rm{{\prime} }}{\rm{{\prime} }}}}\limits^{ \sim }$	J·m/(kg·s)	$\mathop{{v}^{{\rm{{\prime} }}{\rm{{\prime} }}}{E}^{{\rm{{\prime} }}{\rm{{\prime} }}}}\limits^{ \sim }=\bar{\rho {v}^{{\rm{{\prime} }}{\rm{{\prime} }}}{E}^{{\rm{{\prime} }}{\rm{{\prime} }}}}/\bar{\rho }$
Favre-averaged spanwise velocity and total energy fluctuations	favre_wffEff	$\mathop{{w}^{{\rm{{\prime} }}{\rm{{\prime} }}}{E}^{{\rm{{\prime} }}{\rm{{\prime} }}}}\limits^{ \sim }$	J·m/(kg·s)	$\mathop{{w}^{{\rm{{\prime} }}{\rm{{\prime} }}}{E}^{{\rm{{\prime} }}{\rm{{\prime} }}}}\limits^{ \sim }=\bar{\rho {w}^{{\rm{{\prime} }}{\rm{{\prime} }}}{E}^{{\rm{{\prime} }}{\rm{{\prime} }}}}/\bar{\rho }$

## Technical Validation

**Fig. 3:**
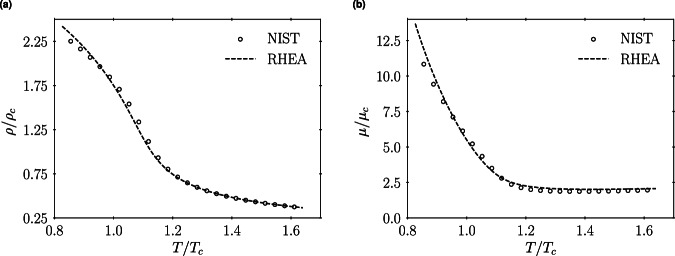
Comparison between NIST data^44^ and RHEA results of density normalized by critical density $\rho /{\rho }_{c}$ (**a**) and dynamic viscosity normalized by critical dynamic viscosity $\mu /{\mu }_{c}$ (**b**) for CO_2_ at $P/{P}_{c}=2$ in the range $T/{T}_{c}\approx 0.8-1.6$.

The computational framework utilized in this work is comprehensively described by Bernades *et al*.^20^. Additionally, the flow solver RHEA^32^ has been extensively validated against canonical flows at ideal-gas and high-pressure transcritical conditions in the works by Abdellatif, Barea, Masclans & Monteiro *et al*.^18,19,22,34^. In particular, the high-pressure thermophysical framework implemented in RHEA^32^, comprising the Peng-Robinson^25^ real-gas equation of state and thermodynamic potentials, as well as the transport coefficients modeled by Chung *et al*.^29,30^, is validated through a comparison with the intermediate pressure scenario of the dataset cases at $P/{P}_{c}=2$. This validation is conducted across a range of temperatures that are available in the reference NIST^44^ database. Figure 3 illustrates that the continuous solution in RHEA, for the temperature range $T/{T}_{c}=0.8-1.6$, closely matches the reference data for density and dynamic viscosity. Notably, the thermophysical model effectively captures the rapid variations in these properties across the pseudo-boiling region at ${T}_{pb}/{T}_{c}\approx 1.1$, which corresponds to a second-order phase transition from supercritical liquid-like to gas-like fluid.

**Fig. 4:**
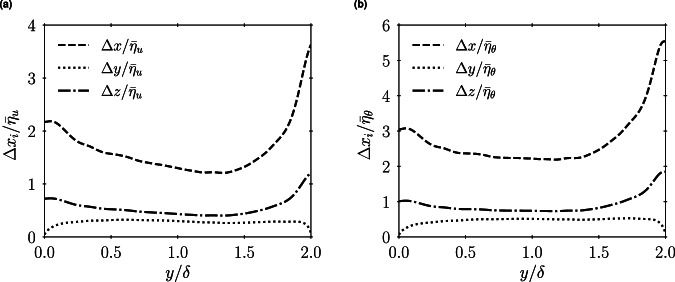
Local mesh resolution in the three directions $\Delta {x}_{i}$ normalized by mean Kolmogorov ${\bar{\eta }}_{u}$ (**a**) and Batchelor ${\bar{\eta }}_{\theta }$ (**b**) scales along the wall-normal direction $y/\delta$.

Additionally, when creating the dataset, a key consideration is achieving adequate mesh resolution in the three spatial directions. In this regard, as shown in Fig. 4, the grid resolution is comparable to the Kolmogorov scale ${\bar{\eta }}_{u}$, which is determined as ${\bar{\eta }}_{u}={[{(\bar{\mu }/\bar{\rho })}^{3}/(\bar{\varepsilon }/\bar{\rho })]}^{\mathrm{1/4}}$ with the turbulent kinetic energy dissipation rate defined as $\bar{{\varepsilon }}=\overline{{\tau }_{ij}{\rm{\partial }}{u}_{i}^{{\prime\prime} }/{\rm{\partial }}{x}_{j}}$, and the Batchelor scale calculated as ${\bar{\eta }}_{\theta }={\bar{\eta }}_{u}/\sqrt{Pr}$. It is important to note that the resolution values fall significantly below the accepted ranges for channel flow with highly temperature-dependent thermophysical properties^45–47^, which suggests that resolutions of $\Delta x < 12\{{\bar{\eta }}_{u},{\bar{\eta }}_{\theta }\}$, $\Delta y < 2\{{\bar{\eta }}_{u},{\bar{\eta }}_{\theta }\}$ and $\Delta z < 6\{{\bar{\eta }}_{u},{\bar{\eta }}_{\theta }\}$ are sufficient for capturing all the relevant flow scales. In particular, for the low Reynolds numbers considered in this database, by utilizing resolutions that are finer than the ones employed by reference research works, like for example Yang *et al*.^48^, it is ensured that even high-intensity wall-shear stress events are resolved with 99% of accuracy. Likewise, Kim *et al*.^49^ mentioned that $\Delta {x}^{+}=10.98$, $\Delta {y}^{+}=0.43-5.93$, $\Delta {z}^{+}=5.63$ with $R{e}_{{\tau }_{cw}}=366$, and $\Delta {x}^{+}=11.58$, $\Delta {y}^{+}=0.46-6.25$, $\Delta {z}^{+}=5.94$ with $R{e}_{{\tau }_{hw}}=386$ is sufficient to ensure third-order convergence of flow statistics. Similarly, Li *et al*.^50^ utilized a mesh similar to the one in this study to investigate up to fourth-order moments of fluctuations for high-pressure transcritical fluid flows with $R{e}_{\tau }=300-1370$.

## Usage Notes

**Fig. 5:**
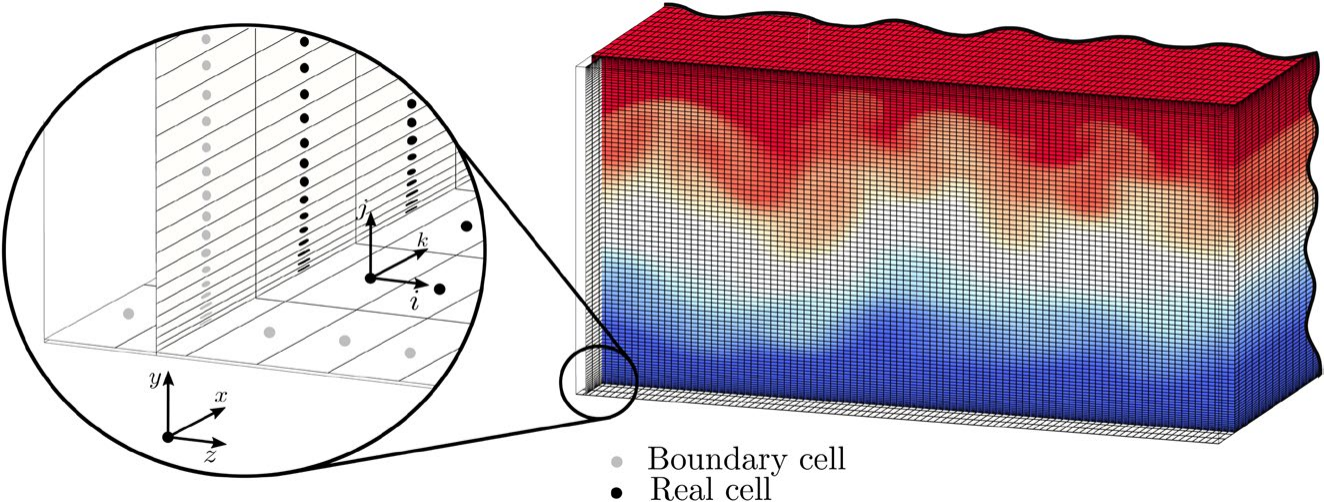
Schematic of a microconfined channel flow mesh domain with detail of the spatial discretization approach.

**Fig. 6:**
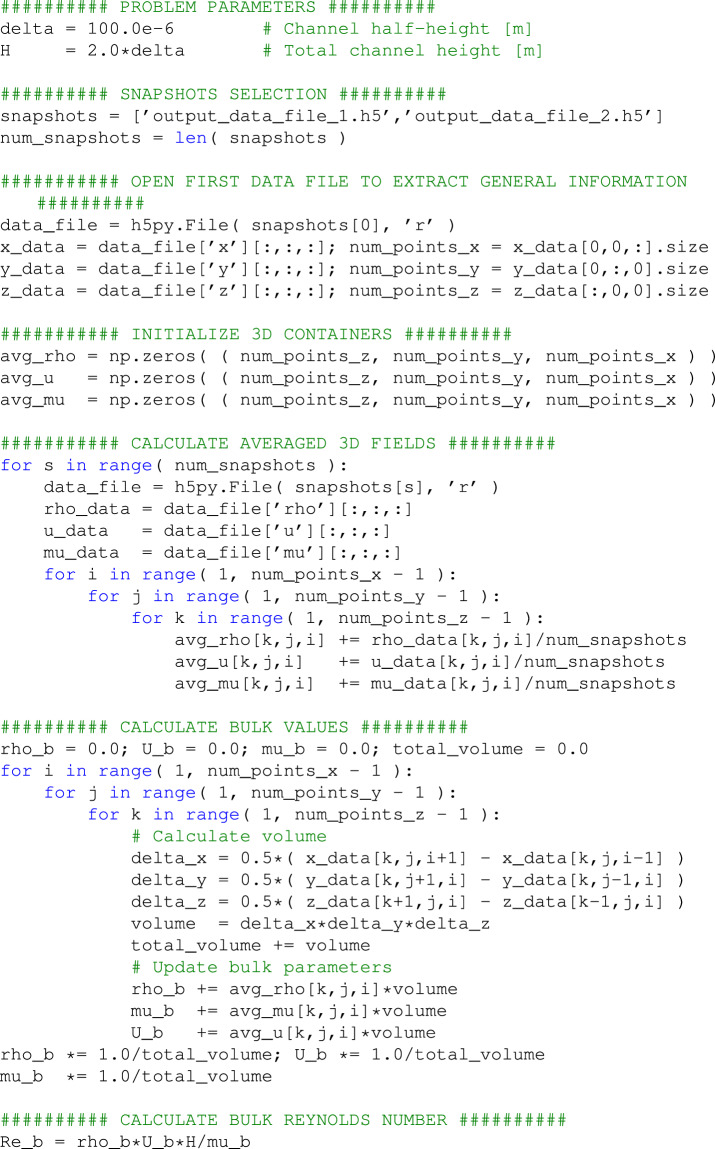
Snippet from the Python code accessing data and calculating the bulk Reynolds number from output data files.

A post-processing script designed to facilitate the output of main variables alongside the computation of derived quantities is available in the main folder. The diligently curated dataset is perfectly tailored for direct use, providing easily accessible quantities extracted from the HDF5 files of each snapshot. Each field within the dataset constitutes a 3D array comprising 98 × 98 × 98 data points. It is worth noting that the initial and final cells in every direction, designated as boundary cells, are reserved for wall calculations. Additionally, users possess the flexibility to navigate the mesh in accordance with their individual computational needs, with the understanding that the mesh coordinates are denoted as (*k*, *j*, *i*), where the *x*, *y*, and *z* coordinates correspond to the *i*, *j*, and *k* directions respectively, as depicted in Fig. 5. An illustrative example of harnessing the dataset to formulate a straightforward computation of the bulk Reynolds number *Re*_*b*_ can be found in Fig. 6. This example serves as a practical demonstration of how to effectively employ various field formats and navigate through the multiple directions of the mesh. In this illustrative case, it is imperative to note that, owing to the implementation of a stretching discretization methodology at non-slip boundaries, the precise computation of the bulk value requires the application of a volume-weighted average to the relevant quantities.

## Data Availability

All source code for RHEA is made available through GitLab as open-source at https://gitlab.com/ProjectRHEA/flowsolverrhea. A release version of the code is available on Zenodo^51^.
